# A DNA repair pathway score predicts survival in human multiple myeloma: the potential for therapeutic strategy

**DOI:** 10.18632/oncotarget.1740

**Published:** 2014-02-24

**Authors:** Alboukadel Kassambara, Claire Gourzones-Dmitriev, Surinder Sahota, Thierry Rème, Jérôme Moreaux, Hartmut Goldschmidt, Angelos Constantinou, Philippe Pasero, Dirk Hose, Bernard Klein

**Affiliations:** ^1^ CHU Montpellier, Institute of Research in Biotherapy, Montpellier, F-34295 FRANCE; ^2^ INSERM, U1040, Montpellier, F-34197 France; ^3^ Cancer Sciences Unit, Faculty of Medicine, University of Southampton, UK; ^4^ Medizinische Klinik V, Universitaetsklinikum Heidelberg, Heidelberg D-69120 GERMANY; ^5^ Institute of Human Genetics, CNRS-UPR1142, Montpellier F-34396 FRANCE; ^6^ Université MONTPELLIER1, UFR Médecine

**Keywords:** DNA repair, Multiple Myeloma, prognosis

## Abstract

DNA repair is critical to resolve extrinsic or intrinsic DNA damage to ensure regulated gene transcription and DNA replication. These pathways control repair of double strand breaks, interstrand crosslinks, and nucleotide lesions occurring on single strands. Distinct DNA repair pathways are highly inter-linked for the fast and optimal DNA repair. A deregulation of DNA repair pathways may maintain and promote genetic instability and drug resistance to genotoxic agents in tumor cells by specific mechanisms that tolerate or rapidly bypass lesions to drive proliferation and abrogate cell death. Multiple Myeloma (MM) is a plasma cell disorder characterized by genetic instability and poor outcome for some patients, in which the compendium of DNA repair pathways has as yet not been assessed for a disease-specific prognostic relevance. We design a DNA repair risk score based on the expression of genes coding for proteins involved in DNA repair in MM cells. From a consensus list of 84 DNA repair genes, 17 had a bad prognostic value and 5 a good prognostic value for both event-free and overall survival of previously-untreated MM patients. The prognostic information provided by these 22 prognostic genes was summed within a global DNA repair score (^DR^Score) to take into account the tight linkage of repair pathways. ^DR^score was strongly predictive for both patients' event free and overall survivals. Also, ^DR^score has the potential to identify MM patients whose tumor cells are dependent on specific DNA repair pathways to design treatments that induce synthetic lethality by exploiting addiction to deregulated DNA repair pathways.

## INTRODUCTION

Multiple myeloma (MM) is a plasma cell disease arising from the malignant transformation of post-follicular B cells and affects 22000 new individuals in the EU or US each year[1]. This disease is characterized by extensive molecular heterogeneity in multiple myeloma cells (MMCs) and diversity in overall survival of patients, which ranges from several months to more than ten years[2-4]. MM can be classified into hyperdiploid MM characterized by chromosome duplication (48-75 chromosomes) in MMCs or non-hyperdiploid MM. Primary translocations involving the immunoglobulin heavy chain locus and recurrent target genes are identified in about 70% of non-hyperdiploid MM and 15% of hyperdiploid MM[5, 6]. Additional molecular defects target various genes, which deregulate the p53 pathway (monoallelic deletion of the *TP53* gene and *TP53* mutations), NK-B pathway (mutations or amplifications), RAS pathway (mutations), or *MYC* pathway (amplification, rare translocations)[7, 8]. These abnormalities may concur to deregulate cell cycle checkpoints and impact on the array of DNA repair pathways[9].

In healthy cells, pleiotropic DNA damage occurs each day due to spontaneous base alterations, exposure to endogenous metabolites or exogenous agents, and errors during DNA replication[10, 11]. Multiple DNA repair proteins function together in order to detect and repair the different types of DNA lesions to avoid cell death from excess DNA damages. There are 6 major DNA repair pathways active in mammalian cells. Base excision repair (BER), nucleotide excision repair (NER) and mismatch repair (MMR) operate on nucleotide lesions occurring on single strands. The BER pathway repairs damaged bases [10] and the MMR pathway targets insertion/deletion loops and mismatches errors during replication[12]. The NER pathway removes bulky lesions, in particular resulting from UV induced DNA damages such as pyrimidine adducts[13]. Two main pathways, homologous recombination (HR) and non-homologous end joining (NHEJ) are involved in DNA double strand breaks (DSBs), which are highly cytotoxic[11]. Finally, proteins involved in the Fanconi Anemia disease (Fanconi anemia [FA] pathway) cooperate with NER and HR pathways to repair interstrand crosslinks (ICLs), which are covalent links between two opposite strands of DNA induced by exposure to chemicals such as bifunctional alkylating agents[14, 15]. The mechanisms of DNA repair have been extensively reviewed recently[11, 16]. They involve briefly DNA lesion recognition, DNA exonuclease, DNA polymerase and DNA ligase activities. DNA repair pathways are highly inter-connected due to the fact that a DNA repair protein can be involved in two or more pathways and that a repair engages several pathways, requiring tight regulatory control in normal cells[11, 16].

A deregulation of these DNA repair pathways could readily promote genetic instability and drug resistance in MMCs by bypassing or accelerating non-accurate DNA repairs to prevent cell death as reviewed recently[9]. Since the MM clone evolves at the genome level as disease progresses, it is highly likely that deregulated DNA repair pathways are implicated in clonal evolution[9, 17, 18]. These pathways are also of particularly relevance for genotoxic drugs used to treat patients with MM, presently doxorubicin, melphalan, cyclophosphamide, and bendamustine[9]. This is again the case for Bortezomib, a proteasome inhibitor and not directly genotoxic, which targets homologous recombination by depleting the pool of free ubiquitin [19]. Consequently, DNA repair pathways in MM are highly relevant to understanding response to the current spectrum of therapeutics agents in clinical use.

In the current study, we investigate the prognostic value of gene expression based scores built to systematically assess genes encompassing the major DNA repair pathways. The data reveals specific patterns of gene expression in MMCs that have prognostic value for both event free and overall survival of newly-diagnosed patients.

## RESULT

### Linking expression levels of DNA repair genes and patient overall survival

A consensus list set of 84 genes coding for the main 6 DNA repair pathways was obtained by review of medline and the current literature of DNA repair pathways (Supplementary Table S2)[11, 16, 20]. The 6 DNA repair pathways were non-homologous end-joining (NHEJ), homologous recombination (HR), Fanconi anemia pathway (FA), nucleotide excision repair (NER), mismatch repair (MMR) and base excision repair (BER). Using the R MaxStat function and Benjamini Hochberg multiple testing correction, 17 out of the 84 genes had bad prognostic value and 5 a good prognostic value for both event-free and overall survivals using the patients of HM cohort (Table 1). These 22 prognostic genes include 5 genes coding for NHEJ pathway (3 bad: *WHSC1, RIF1 and XRCC5(KU80);* 2 good*: PNKP and POLL)*, 6 genes for HR (5 bad: *EXO1, BLM, RPA3, RAD51 and MRE11A;* 1 good*: ATM*), 3 bad genes for FA (*RMI1, FANCI and FANCA*), 8 genes for NER (6 bad: *PCNA, RPA3, LIG3, POLD3, ERCC4 and POLD1; 2 good: ERCC1 and ERCC5*), 2 bad genes for MMR (*EXO1* and *MSH2*) and 1 bad gene for BER (*LIG3*) pathways.

**Table 1:** Identification of DNA repair genes whose expression is associated with patients' prognostic value using HM cohort Out of the 84 DNA repair genes, 22 had prognostic value for both event-free survival (EFS) and overall survival (OS) using R MaxStat function. Genes are ranked according to the FDR of log-rank test for overall survival. FDR: false discovery rate; HR: hazard ratio.

				Overall Survival			Event Free Survival		
Probe set	Gene Name	DNA repair pathway	Prognostic value	Maxstat cut-point	FDR	HR	Maxstat cut-point	FDR	HR
218979_at	RMI1	FA	BAD	1356	1.E-04	5	1353	6.E-04	3.0
201202_at	PCNA	NER	BAD	3703	1.E-04	4.5	2446	9.E-04	2.2
222777_s_at	WHSC1/MMSET	NHEJ	BAD	1506	3.E-04	3.7	918	3.E-05	2.8
204603_at	EXO1	HR/MMR	BAD	295	8.E-04	3.9	48	4.E-02	1.8
213007_at	FANCI	FA	BAD	648	9.E-04	3.5	179	1.E-02	2.4
226503_at	RIF1	NHEJ	BAD	1273	2.E-03	3.2	1273	2.E-03	2.3
205733_at	BLM	HR	BAD	1450	4.E-03	2.9	1495	2.E-02	1.8
209421_at	MSH2	MMR	BAD	608	3.E-02	2.7	352	3.E-02	1.6
209507_at	RPA3	HR/NER	BAD	3902	3.E-02	3.2	3836	5.E-04	3.1
204123_at	LIG3	NER/BER	BAD	502	3.E-02	2.6	435	4.E-03	2.0
212836_at	POLD3	NER	BAD	262	3.E-02	6.3	247	2.E-02	2.1
205024_s_at	RAD51	HR	BAD	641	3.E-02	2.8	514	5.E-02	1.7
208642_s_at	XRCC5/ KU80	NHEJ	BAD	7702	4.E-02	2.9	7626	2.E-03	2.5
235215_at	ERCC4	NER	BAD	1103	4.E-02	2.5	873	1.E-03	2.2
203805_s_at	FANCA	FA	BAD	271	4.E-02	2.2	140	1.E-02	2.4
203422_at	POLD1	NER	BAD	762	5.E-02	2.4	714	1.E-02	2.0
205395_s_at	MRE11A	HR	BAD	544	5.E-02	2.1	542	1.E-02	1.8
203720_s_at	ERCC1	NER	GOOD	1885	4.E-02	0.4	3184	2.E-02	0.4
218961_s_at	PNKP	NHEJ	GOOD	1473	4.E-02	0.4	1346	2.E-03	0.5
221049_s_at	POLL	NHEJ	GOOD	101	5.E-02	0.3	42	5.E-03	0.5
202414_at	ERCC5	NER	GOOD	645	5.E-02	0.5	695	5.E-03	0.5
212672_at	ATM	HR	GOOD	1570	5.E-02	0.5	1575	3.E-02	0.6

### Building a global DNA repair pathway score (^DR^score) for predicting patients' survival

**Figure 1:**
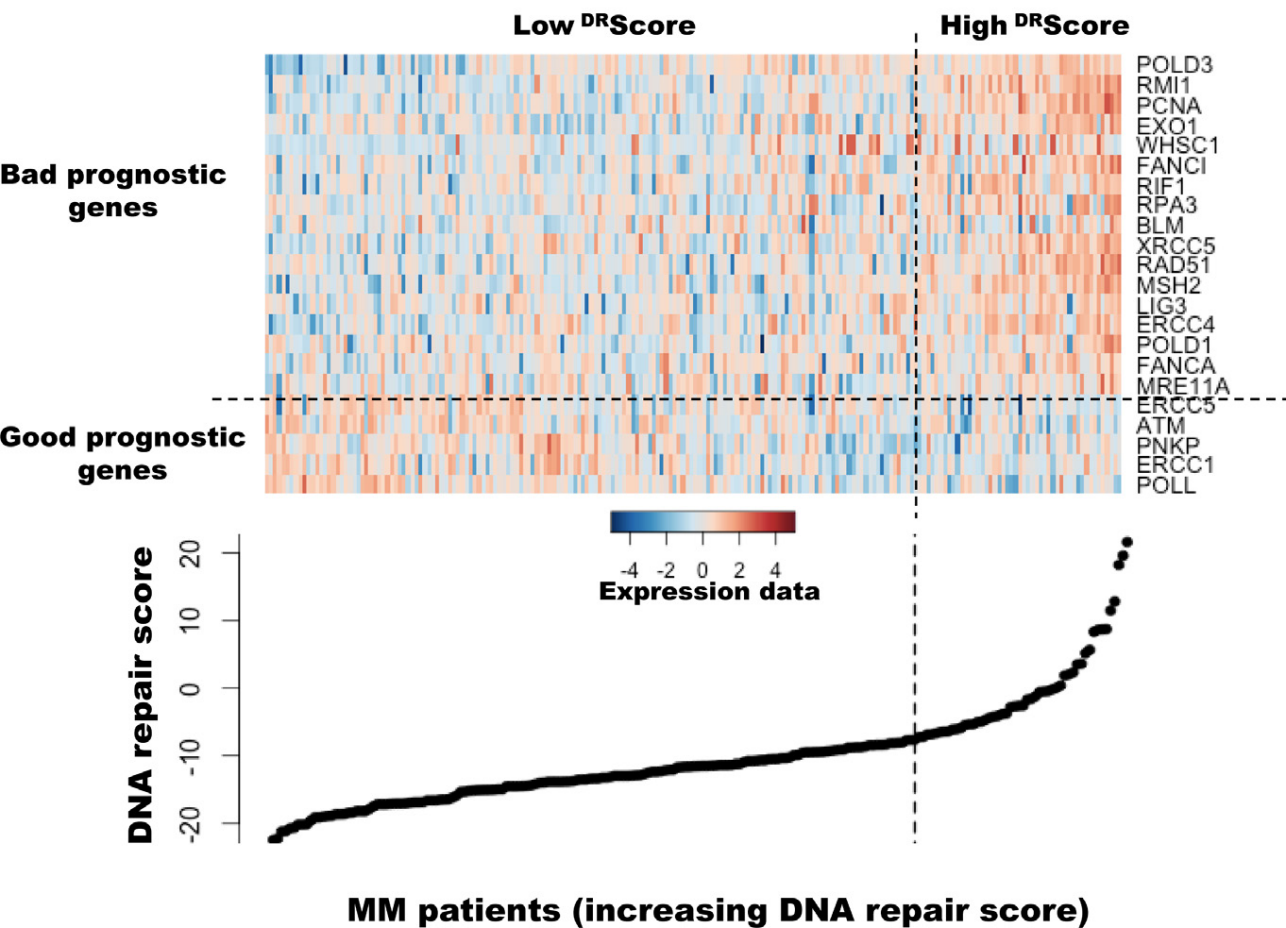
Heatmap of the gene expression signals of the 22 genes used to build DNA repair score in myeloma cells of 206 previously untreated patients The signals of the 22 genes in MMCs of 206 patients, ordered by increasing DRScore, are displayed from low (deep blue) to high (deep red) expression.

**Figure 2:**
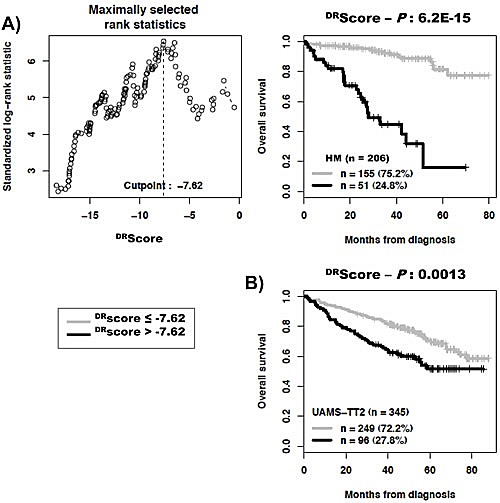
Building a DNA repair score (^DR^score) for predicting overall survival A. The prognostic information provided by the 22 DNA repair genes was summed within a ^DR^score as defined in the Materials and Methods. Patients of the HM cohort were ranked according to increased ^DR^score and a maximum difference in overall survival (OS) was obtained with a ^DR^score = −7.62 splitting patients in a high risk (24.8%) and low risk (75.2%) groups. B. Validation of ^DR^score using the UAMS-TT2 cohort.

**Figure 3:**
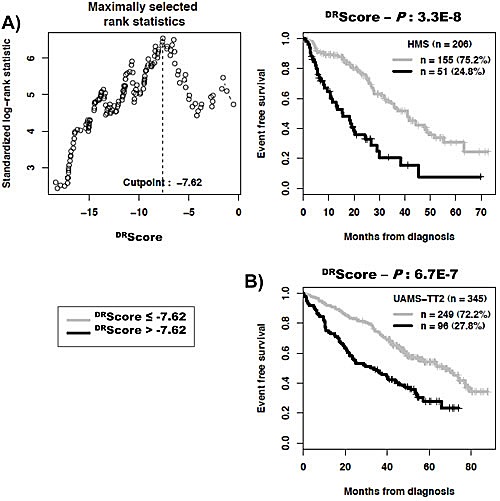
Link between DNA repair pathway score (^DR^score) and patients event-free survival (EFS) The prognostic information provided by the 22 DNA repair genes was summed within a ^DR^score as defined in the Materials and Methods. The link between ^DR^score and patients EFS has been investigated using ^DR^score cut-off of −7.62 splitting patients in a high risk (27%) and low risk (73%) groups.

As DNA repair pathways are tightly linked, the prognostic information provided by the 22 prognostic DNA repair genes was summed within a global DNA repair pathway (^DR^score) as indicated in the Materials and Methods. The variation of ^DR^score in malignant plasma cell populations is shown in Figure 1. The Maxstat statistic test cuts the HM-patient cohort within 2 groups: 24.8% of patients with a ^DR^score > −7.62 with a median OAS of 27.9 months and 75.2% of patients with a ^DR^score ≤ −7.62 with a median OAS not-reached (*P* = 6.2E^−15^, Figure 2A). The ^DR^score could also predict for EFS (Figure 3A). The high-risk ^DR^score group had a median EFS of 15.7 months and the low-risk ^DR^score one a median EFS of 41.1 months (*P* = 3.3E^−8^, Figure 3A). ^DR^score was also prognostic for the UAMS-TT2 cohort of 345 patients. Using the cutoff of −7.62 defined on the HM cohort, 27.8% of UAMS-TT2 cohort patients were identified as high-risk ^DR^score (*P* =.001, Figure 2B). The median OAS was not reached in this cohort but at 50 months of follow-up, 85% of patients in the low risk ^DR^score group are alive compared to only 65% in the high-risk group (*P* =.001, Figure 2B). Patients of the high-risk group had a median EFS decreased about 2-fold compared to those of the low risk-group (32.3 months vs. 69.6 months, *P* =.001, Figure 3B).

### Cox univariate and multivariate analysis of ^DR^score for overall survival compared with the other prognostic clinical factors

Prognostic value for overall survival of ^DR^score was compared with usual prognostic factors - ISS, t(4;14), del17p - or 4 published GEP-based risk scores, UAMS-HRS[21], IFM score[22], GPI[23] and RS score[4]. Using univariate Cox analysis on HM cohort, all these factors had prognostic value and the ^DR^score had the higher hazard ratio (Table 2A). Using multivariate Cox analysis, ^DR^score, RS score, t(4;14), ISS, 2m and albumin kept prognostic value (Tables 2 B and 2C). Univariate cox analysis on UAMS-TT2 cohort showed that UAMS-HRS had the higher hazard ratio, followed by del17p, t(4;14), ^DR^score, GPI and IFM score, ISS, 2m and albumin (Table 2A). Using multivariate Cox analysis, ^DR^score remained significant when tested together with IFM score, RS score, t(4;14), del17p, ISS, 2m and albumin (Table 2C). However, it failed to be significant when tested together with UAMS-HRS, likely due to overfitting since UAMS-HRS was built using UAMS-TT2 cohort.

**Table 2:** Cox univariate and multivariate analysis of overall survival in HM and UAMS-TT2 patients' cohorts A) Cox univariate analysis of overall survival. The prognostic factors were tested as single variable. B) Cox multivariate analysis of overall survival. The ^DR^score were tested together with each of the prognostic factors. C) Cox multivariate analysis of overall survival using all prognostic factors together. Hazard ratios (HR) and *P*-values are shown. * A spike expression of *MMSET* gene was used a surrogate marker for t(4;14) as described[27]. NS, Not Significant at a 5% threshold; GPI, gene expression based proliferation index; ISS, International Staging System; UAMS-HRS, high-risk score from UAMS; IFM, Intergroupe Francophone du Myelome.

A	Univariate Cox analysis - Overall survival					B	Multivariate Cox analysis - Overall survival			
	HMS		UAMS - TT2				HMS		UAMS - TT2	
	HR	P	HR	P			HR	P	HR	P
^DR^Score	9	4.0E-11	1.9	1.6E-03		^DR^Score	10	1.3E-10	1.1	NS
UAMS HRS	2.4	1.4E-02	4.7	4.8E-13		UAMS HRS	0.75	NS	4.4	1.8E-09
IFM score	2.5	1.9E-02	1.8	4.0E-03		^DR^Score	9	2.1E-10	1.6	1.8E-02
GPI	2.6	1.6E-04	1.8	2.2E-04		IFM score	1	NS	1.5	4.8E-02
RS score	4.2	3.3E-09	1.9	1.0E-05		^DR^Score	8.2	6.4E-08	1.4	NS
t(4;14)*	3.3	4.7E-04	2.2	3.2E-04		GPI	1.2	NS	1.5	1.5E-02
del17p	3.4	2.0E-02	2.5	3.7E-04		^DR^Score	4.9	2.4E-04	1.2	NS
ISS	2	9.7E-04	1.6	5.5E-05		RS score	1.9	2.3E-02	1.8	1.5E-03
B2M	1.1	4.2E-05	1.1	4.9E-08		^DR^Score	8.2	8.4E-10	1.7	1.1E-02
Alb	0.47	1.4E-02	0.94	1.2E-04		t(4;14)*	2.2	3.0E-02	2	2.9E-03
						^DR^Score	11	1.8E-11	1.7	9.3E-03
						del17p	2.8	NS	2.1	3.2E-03
C	Multivariate Cox analysis - Overall survival					^DR^Score	8.8	1.6E-10	1.7	1.1E-02
	HMS		UAMS - TT2			ISS	1.8	3.3E-03	1.5	3.4E-04
	HR	P	HR	P		^DR^Score	9.1	5.8E-11	1.6	1.4E-02
^DR^Score	18	2.1E-07	0.64	NS		B2M	1.1	1.0E-04	1.1	2.1E-06
UAMS HRS	0.55	NS	3.5	6.8E-06		^DR^Score	9.2	1.8E-11	1.7	8.8E-03
IFM score	0.34	NS	0.95	NS		Alb	0.44	8.2E-03	0.95	6.6E-04
GPI	0.86	NS	1.3	NS						
RS score	1.1	NS	1.1	NS						
t(4;14)*	2.7	3.8E-02	2.4	7.5E-04						
del17p	3.1	NS	2.6	2.6E-04						
ISS	2.9	2.0E-04	1.5	1.3E-03						

### Cox univariate and multivariate analysis of ^DR^score for event free survival compared with the other prognostic clinical factors

Prognostic value for event free survival of ^DR^score was compared with the other prognostic factors mentioned above. Using univariate Cox analysis on HM cohort, all these factors were significantly associated with patients' event free survival. The deletion 17p had the higher hazard ratio followed by ^DR^score (Table 3A). Using multivariate Cox analysis, ^DR^score, RS score, t(4;14), del17p, ISS, 2m and albumin, kept prognostic value. Univariate cox analysis on UAMS-TT2 cohort showed that UAMS-HRS had the higher hazard ratio followed by t(4;14) and ^DR^score. Using multivariate Cox analysis, ^DR^score remained an independent prognostic factor for EFS when tested together with UAMS-HRS, IFM score, RS score, t(4;14), del17p, ISS, 2m and albumin (Table 3C). GPI score failed to be significant.

**Table 3:** Cox univariate and multivariate analysis of event free survival (EFS) in HM and UAMS-TT2 patients' cohorts A) Cox univariate analysis of EFS. The prognostic factors were tested as single variable. B) Cox multivariate analysis of EFS. The ^DR^score were tested together with each of the prognostic factors. C) Cox multivariate analysis of EFS using all prognostic factors together. Hazard ratios (HR) and *P*-values are shown. * A spike expression of *MMSET* gene was used a surrogate marker for t(4;14) as described[27]. NS, Not Significant at a 5% threshold; GPI, gene expression based proliferation index; ISS, International Staging System; UAMS-HRS, high-risk score from UAMS; IFM, Intergroupe Francophone du Myelome.

A	Univariate Cox analysis - Event free survival					B	Multivariate Cox analysis - Event free survival			
	HMS		UAMS - TT2				HMS		UAMS - TT2	
	HR	P	HR	P			HR	P	HR	P
^DR^Score	3.1	1.4E-07	2.1	1.2E-06		^DR^Score	2.9	9.7E-06	1.6	6.6E-03
UAMS HRS	1.9	5.3E-03	3.5	3.8E-11		UAMS HRS	1.2	NS	2.7	2.6E-06
IFM score	1.9	1.6E-02	1.9	5.2E-05		^DR^Score	2.9	2.7E-06	1.9	1.3E-04
GPI	1.8	3.3E-04	1.5	4.1E-04		IFM score	1.2	NS	1.6	5.8E-03
RS score	2	2.7E-06	1.7	9.3E-06		^DR^Score	2.6	1.3E-04	1.9	6.5E-04
t(4;14)*	3.1	2.9E-05	2.4	9.2E-07		GPI	1.3	NS	1.2	NS
del17p	3.4	2.5E-03	2	1.3E-03		^DR^Score	2.1	6.9E-03	1.7	7.2E-03
ISS	1.3	2.3E-02	1.5	7.2E-07		RS score	1.5	4.3E-02	1.4	3.5E-02
B2M	1	6.7E-03	1.1	6.7E-10		^DR^Score	2.7	1.0E-05	2	1.4E-05
Alb	0.68	5.0E-02	0.96	2.8E-03		t(4;14)*	2.3	3.6E-03	2.2	1.5E-05
						^DR^Score	3.3	7.6E-08	2	1.0E-05
						del17p	2.7	1.4E-02	1.7	1.6E-02
C	Multivariate Cox analysis - Event free survival					^DR^Score	3.4	2.7E-08	1.9	2.9E-05
	HMS		UAMS - TT2			ISS	1.3	4.8E-02	1.5	1.5E-05
	HR	P	HR	P		^DR^Score	3.3	4.9E-08	1.9	6.6E-05
^DR^Score	2.4	8.7E-03	1.1	NS		B2M	1	4.1E-02	1.1	1.7E-07
UAMS HRS	1	NS	1.8	1.4E-02		^DR^Score	3.1	1.1E-07	2	7.9E-06
IFM score	1.1	NS	1.3	NS		Alb	0.67	4.9E-02	0.97	1.9E-02
GPI	1.1	NS	1.3	NS						
RS score	1.1	NS	0.94	NS						
t(4;14)*	2.3	9.2E-03	2.6	8.4E-06						
del17p	2.7	2.6E-02	2.1	1.1E-03						
ISS	1.4	3.8E-02	1.5	3.2E-05						

### Link of ^DR^score with patients' clinical and genetic parameters

The frequencies of patients with high lactate dehydrogenase or C-reactive protein levels were significantly increased in patients with high risk ^DR^score (*P* ≤.05, Table 4). Others clinical data – age, β2m, albumin, hemoglobin, ISS staging, Salmon-Durie staging, light or heavy chain isotype and occurrence of bone lesions - were not significantly different between the 2 ^DR^score groups. The frequency of patients with t(4;14),1q21, del17p or del13 was significantly increased in the high risk ^DR^score group (*P* ≤.05, Table 5).

Table 4Clinical characteristics of patients in the 2 groups defined by ^DR^scoreThe 206 previously-untreated patients of the HM cohort were treated at the university hospitals of Heidelberg and Montpellier. Patients were separated in 2 groups: low-risk (^DR^score ≤ −7.62) and high-risk (^DR^score > −7.62) ^DR^score groups. Data are the percentages of patients within these 2 groups with the indicated clinical or biological parameters. When the percentages were different with a chisquare test (P ≤.05), data are shown in bold.

**Table:** 

Categories	^DR^Score groups	
	^DR^Score ≤ −7.62 (n = 155)	^DR^Score > −7.62 (n = 51)
	% of patients in each group	
Age >= 65 yr	17%	25%
IgA subtype	21%	27%
Kappa light chain	66%	55%
Lambda light chain	30%	43%
Non-secreting	2%	2%
B2M<= 3.5 mg/ml	66%	55%
B2M> 5.5 mg/ml	15%	20%
LDH>= 240 IU/liter	20%	32%
Albumin < 35 g/liter	32%	31%
Hemoglobin< 10 g/dl	26%	37%
C-reactive protein >= 5 mg/liter	31%	51%
Bone lesions		
0: normal bone structure	22%	17%
1: osteopenie / osteoporosis	32%	29%
2: osteolyse [1-3]	6%	10%
3: major structural damage [>3]	39%	45%

**Table:** 

	^DR^Score ≤ −7.62(n = 155)			^DR^Score > −7.62 (n = 51)		
Staging	I	II	III	I	II	III
Salmon-Durie	12%	15%	73%	8%	16%	76%
ISS	51%	34%	15%	39%	41%	20%

**Table 5:** Link of ^DR^score with patients' genetic abnormalities Interphase-FISH-analysis was performed on CD138-purified plasma cells of 153 to 169 patients of the HM series, depending on the gene abnormality Patients were separated in two groups according to DRscore (low-risk and high-risk groups). Data are the percentages of patients within these 2 groups with the biological parameters. When the percentages were different with a chisquare test (P ≤.05), data are shown in bold.

	^DR^score ≤ −7.62	^DR^score > −7.62
t(11;14)+(n = 27)	17%	15%
t(11;14)-(n = 140)	83%	85%
t(4;14)+(n = 28)	13%	28%
t(4;14)-(n = 137)	87%	72%
1q21+(n = 62)	33%	59%
1q21-(n = 91)	67%	41%
del13+(n = 91)	45%	77%
del13-(n = 78)	55%	23%
del17+(n = 27)	14%	24%
del17-(n = 132)	85%	76%

### Prognostic value of scores integrating genes coding for either NHEJ, HR, FA, NER or MMR DNA repair pathways

**Figure 4:**
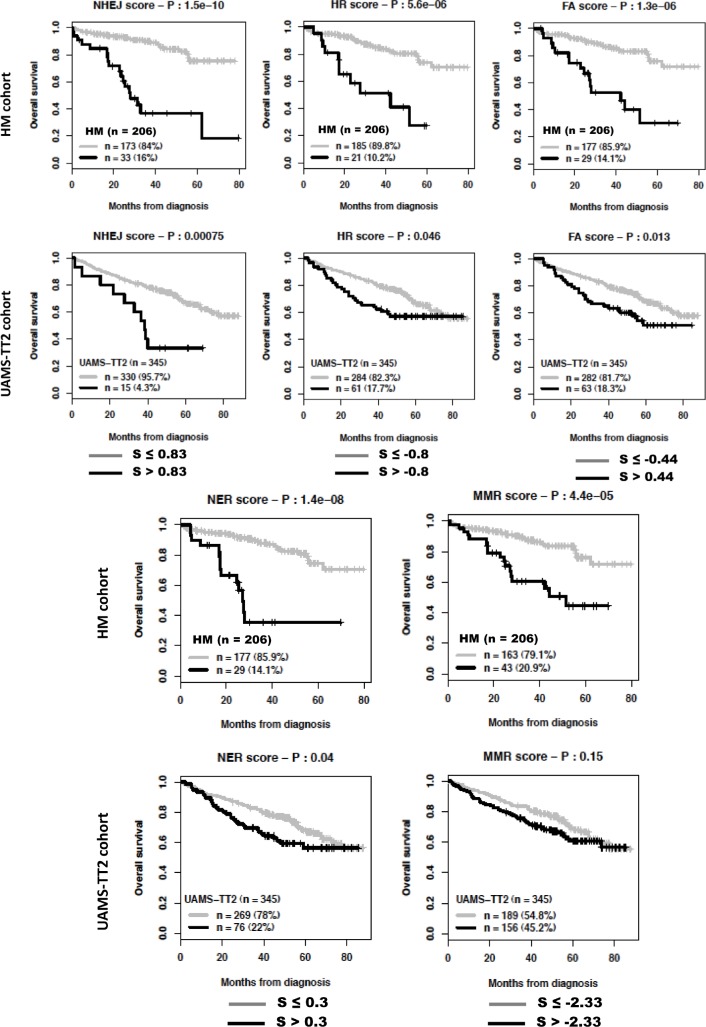
Prognostic value of NHEJ, HR, FA, NER and MMR DNA repair pathways For a given pathway, a prognostic score was calculated, as described in the Materials and Methods, by integrating the prognostic information provided by the prognostic genes coding for proteins involved in the pathway. NHEJ: non-homologous end joining; HR: homologous recombination; FA: fanconi anemia pathway; NER: nucleotide excision repair; MMR: mismatch repair; S: score..

The global DNA repair score described above incorporates all prognostic genes coding for the various DNA repair pathways. Despite a reduced number of prognostic genes coding for a specific pathway (Table 1), we looked whether scores built using the same methodology as the global DNA repair score and specific for a pathway could have prognostic value. Using maxstat analysis for overall survival, NHEJ, HR, FANC or NER scores were significantly associated with high-risk myeloma in the 2 independent patients' cohorts, HM and UAMS-TT2 (Figure 4). MMR score had only prognostic value for the HM cohort and BER score was not considered since it comprises one prognostic gene only.

## DISCUSSION

By selecting genes coding for proteins involved in repair of DNA damages, we have built a DNA repair score assembling genes encoding for the various DNA repair pathways. This is particularly relevant since these various pathways are tightly linked and inter-dependent to repair DNA [10, 11]. The ^DR^score is predictive for both event free and overall survival as evaluated in two independent cohorts of patients with MM. It remained an independent prognostic factor when tested together with known molecular prognostic factors such as previously-published GEP-based risk scores, t(4;14), del17p and with standard clinical prognostic factors, ISS, 2m and albumin in two independent large patients' cohorts. The UAMS-TT2 cohort is particularly relevant in comparing the prognostic value of these factors, excluding UAMS-HRS, as ^DR^score was not designed on this cohort, which avoids overfitting. Of note, prognostic scores assembling genes coding for a specific DNA repair pathway can be also built, despite the reduced number of prognostic genes for each pathway.

DNA repair pathways are deregulated in many MM patients and could provide adaptive mechanisms to trigger drug resistance[9]. Novel compounds targeting DNA repair pathways are being clinically evaluated in patients with cancer inducing synthetic lethality[16]. The principle of synthetic lethality is that tumor cells have deregulated cell cycle and/or DNA repair by inactivating some pathways, in particular the p53 pathway, making their survival dependent on remaining pathways. Targeting these remaining pathways will make cells unable to repair DNA damages, complete cell cycle or gene transcription, and bring them to death[16, 34-37]. The current prognostic scores integrating genes coding for NHEJ, HR, FA NER, or MMR pathways could be of interest to identify patients with MM who could benefit from inhibitors targeting key component in these pathways. In particular inhibitors to DNA-PKs (NHEJ), RAD51 (HR), PARP1/2 (HR, altNHEJ, BER), CHK2 (HR, altNHEJ), CHK1 (HR, NER) are currently under clinical investigation in various cancers[16, 34]. A clinical trial investigating the efficacy of PARP1/2 inhibitors in patients with MM resistant to proteasome inhibitors is under development[38]. Indeed, proteasome inhibitors block Fanconi anemia and homologous recombination pathways, rendering MM cells addict on BER initiated by PARP1/2-mediated Poly(ADP-ribosyl)ation of proteins[38]. It is of major interest to look for whether the ^DR^score in MMCs prior treatment could predict for the response of patients to DNA repair inhibitors. In this case, this ^DR^score will be of use in stratifying MM patients and exploiting the addiction of tumor cells to a specific DNA repair pathway.

## METHODS

### Patient samples and gene expression data

Multiple Myeloma cells (MMCs) were purified from the 206 patients with newly-diagnosed MM after written informed consent was given at the University hospitals of Heidelberg (Germany) or Montpellier (France) as described[24]. Clinical characteristics of the HM cohort are provided in Supplementary Table S1. The study was approved by the ethics boards of the University Hospitals of Heidelberg and Montpellier. Gene expression profiling (GEP) of purified MMCs was assayed using Affymetrix U133 2.0 plus microarrays as described[25] and data normalized using the MAS5 Affymetrix algorithm with a scaling factor of 500. The.CEL and MAS5 files are deposited in the ArrayExpress public database (http://www.ebi.ac.uk/arrayexpress/) under accession number E-MTAB-362. We also used publicly available MAS5 normalized GEP data (GEO, http://www.ncbi.nlm.nih.gov/geo/, accession number GSE2658) from purified MMCs of a cohort of 345 patients treated with total therapy 2 protocol (UAMS-TT2 cohort) at the University of Arkansas for Medical Sciences (UAMS, Little Rock, USA) [26]. As iFISH data were not available for UAMS-TT2 patients, t(4;14) translocation was evaluated using MMSET spike expression [27] and del17p13 surrogated by the level of TP53 [28].

### Statistical analysis

Affymetrix gene expression data were normalized using MAS5 Affymetrix algorithm with a scaling factor of 500. The statistical significance of differences in overall survival between groups of patients was calculated by the log-rank test. Multivariate analysis was performed using the Cox proportional hazards model. Survival curves were plotted using the Kaplan-Meier method. All these analyses have been done with R.2.10.1 (http://www.r-project.org/) and bioconductor version 2.5[29, 30]. Gene annotation and networks were generated through the use of Ingenuity Pathways Analysis (Ingenuity^®^ Systems, Redwood City, CA).

### Construction of a DNA Repair Pathway-Focused Score

A consensus list set of 84 genes coding for the main 6 DNA repair pathways has been obtained by review of Medline and the current knowledge of DNA repair pathways [11, 16, 20](Supplementary Table S2). The prognostic value of each of the 84 genes was computed using maximally selected rank test from R package MaxStat (http://cran.r-project.org/web/packages/maxstat/index.html) on HM patient cohort and Benjamini Hochberg multiple testing correction, yielding to 22 genes whose expression values was significantly (P ≤.05) associated with both event-free (EFS) and overall survival (OS). A DNA repair pathway score (termed DRscore) was built to group the prognostic information of these 22 genes within one parameter using a methodology which was proven as powerful in building various gene expression based risk scores[4, 31-33]. For each of the 22 DNA repair genes, the odd ratio of the Cox analysis on the HM cohort were determined with R MaxStat package, and for each patient, these odd ratios were weighted by +1 if the patient's gene expression is above the Maxstat cutoff, and −1 if below or equal this cutoff. The DRscore of a given patient was the sum of these weighted odd ratios for the 22 prognostic genes. DRscore ranges from −22.45 to +21.59 and the higher the DRscore is, the worse the prognosis is. Patients from the same cohort were ranked according to increased DRscore and for a given value S, the difference in overall survival of patients with a DRscore ≤ S or > S was computed, making it possible to define the DRscore value with a maximum difference in survival using maximally selected rank test from R package MaxStat.

## SUPPLEMENTARY TABLES


